# Amblyopia in 2026: A State-of-the-Art Review of Multidimensional Phenotyping, Response Heterogeneity, and Clinical Considerations

**DOI:** 10.3390/brainsci16050467

**Published:** 2026-04-27

**Authors:** Danjela Ibrahimi, José R. García-Martínez

**Affiliations:** 1 Facultad de Medicina, Universidad Autónoma de Querétaro, Santiago de Querétaro 76010, Mexico; 2 Brain Vision & Learning Center, Misión de Capistrano 117, Juriquilla, Santiago de Querétaro 76226, Mexico; 3 Facultad de Ingeniería Mecánica y Eléctrica, Universidad Veracruzana, Poza Rica 93390, Mexico

**Keywords:** amblyopia, interocular suppression, binocular function, clinical phenotyping, COSAMS, functional outcomes, graded optical penalization, digital therapeutics, vision therapy, orthoptics

## Abstract

Amblyopia is increasingly conceptualized as a neurodevelopmental visual disorder that often arises from discordant binocular visual experience during early life and is associated with abnormal binocular interactions, interocular suppression, orientation-dependent developmental abnormalities in selected refractive phenotypes, and experience-dependent plasticity, consistent with a distributed-network perspective rather than a purely monocular acuity deficit. We performed a structured state-of-the-art narrative synthesis of peer-reviewed reviews, randomized controlled trials, and key mechanistic human studies indexed in PubMed/MEDLINE, Web of Science, and Scopus (1 January 2016–28 February 2026; last search 28 February 2026), prioritizing recent evidence from 2021–2026. Literature supports consideration of clinically trackable constructs beyond best-corrected visual acuity (BCVA), including quantified suppression/imbalance, binocular function, and functionally meaningful outcomes such as reading-related limitation and broader functional impact. Across established and emerging intervention classes, treatment effects are heterogeneous across ages and etiologies. Evidence is strongest for conventional penalization and selected active training-based approaches, whereas newer protocol-standardized approaches remain investigational and require prospective evaluation with transparent exposure/dose reporting. Based on these findings, we outline a clinically oriented, core outcome set for amblyopia and strabismus (COSAMS)-aligned framework that combines quantified binocular imbalance with multidimensional phenotyping and a hypothesis-driven, prospectively testable therapeutic model intended to structure (not replace) clinical decision-making. Priorities for precision-oriented amblyopia care include standardization of suppression metrics, adoption of core outcome sets, transparent reporting of ‘not measurable’ outcomes and missingness, and prospective validation of phenotype-driven, prediction-ready frameworks.

## 1. Introduction

Amblyopia is a developmental visual disorder characterized by reduced best-corrected visual acuity (BCVA) affecting one or both eyes that is not fully explained by identifiable structural ocular abnormalities [1,2]. It arises from abnormal visual experience during early development caused by discordant binocular input and/or chronic retinal blur, most commonly associated with refractive causes (including anisometropia, bilateral high refractive error (isoametropia), and clinically significant astigmatism), strabismus, or visual deprivation [1,2,3]. If not effectively addressed during sensitive developmental periods, these conditions may lead to persistent visual impairment beyond childhood [1,4]. From a public health perspective, a systematic review and meta-analysis estimated approximately 99.2 million people with amblyopia worldwide in 2019, with projections increasing to 221.9 million by 2040, underscoring the importance of effective screening and treatment strategies across regions and age groups [5].

Although BCVA is a central clinical endpoint, contemporary syntheses increasingly frame amblyopia as more than an isolated monocular acuity deficit, emphasizing its neurodevelopmental basis and the role of binocular dysfunction in the clinical phenotype [1,4,6]. Abnormal binocular interactions—including interocular suppression and binocular imbalance—are, therefore, treated as mechanistically relevant constructs for phenotyping and for binocularly oriented rehabilitation approaches [1,6]. Importantly, suppression is not only a qualitative clinical descriptor; quantitative paradigms have been developed to measure interocular suppression in children, supporting its use as a measurable dimension in clinical research and potentially in monitoring frameworks [7].

Beyond acuity, functional limitations are increasingly recognized. Clinical evidence links amblyopia with slower reading performance, supporting the inclusion of functionally meaningful endpoints alongside BCVA when evaluating therapeutic benefit [8]. Randomized evidence also indicates that structured active paradigms—tested as patching alone versus patching combined with protocolized vision therapy, and versus monocular perceptual learning—can improve visual outcomes under defined protocols [9]. Higher-order visual processing differences have also been quantified, including altered visual attention and visual search performance in children with amblyopia [10], and impaired fine motor skills, slower reading speed, and reduced self-reported quality of life in adults with amblyopia and/or strabismus [11].

From a treatment standpoint, conventional penalization (patching and/or pharmacologic penalization) remains foundational. A Cochrane systematic review concludes that both conventional occlusion and atropine penalization improve amblyopic-eye visual acuity, with atropine appearing as effective as occlusion overall (with variability across trials) [12].

Across occlusion regimens, a meta-analysis comparing part-time and full-time occlusion found no statistically significant difference overall, supporting the effectiveness of part-time occlusion regimens within the ranges evaluated across trials [13]. Over the last decade, randomized trials have expanded the evidence base beyond penalization to binocular/dichoptic and digital approaches, including binocular iPad game paradigms [14,15], and more recent home-based platforms delivering dichoptic content evaluated against patching and/or control comparators (including optical-only in selected trials) under protocol-defined dosing and endpoints [16,17,18,19,20]. Additional pediatric randomized trial evidence in younger cohorts (4–6 years) reported greater amblyopic-eye visual acuity (VA) improvement at 4 weeks with binocular Dig Rush compared with spectacles alone, whereas between-group differences were less clear by 8 weeks [21]. Moreover, randomized evidence from dichoptic virtual reality paradigms has been reported under structured protocols, supporting further evaluation with careful attention to dosing and endpoint selection [22].

High-level syntheses comparing binocular/dichoptic interventions with standard occlusion/penalization or monocular training report small and inconsistent between-group differences in amblyopic-eye BCVA and binocular outcomes, with substantial heterogeneity influenced by trial selection, treatment design, adherence, and outcome definitions [23,24,25,26,27]. Table 1 is added to illustrate selected (non-exhaustive) randomized and synthesis-level evidence for binocular treatment.

## 2. Methods

We conducted a structured state-of-the-art narrative review focused on neurodevelopmental and neurobiological mechanisms of amblyopia, emphasizing binocular imbalance, interocular suppression, distributed network alterations, and experience-dependent plasticity, with translational links to clinical phenotyping and response heterogeneity.

All references were restricted to peer-reviewed sources, and DOIs are reported in the reference list when verifiable from publisher records and/or indexed bibliographic databases. This approach was selected to integrate heterogeneous evidence streams (mechanistic neuroscience, psychophysics, neuroimaging, and randomized clinical trials) for which quantitative pooling is frequently constrained by methodological and outcome heterogeneity.

We searched PubMed/MEDLINE, Web of Science Core Collection, and Scopus for eligible literature published from 1 January 2016 to 28 February 2026 (date of last search). During screening and synthesis, we prioritized more recent evidence from 2021–2026, particularly on binocular/dichoptic and digital interventions, suppression quantification, COSAMS/core outcome standardization, and functional outcomes beyond BCVA. Search strings were constructed around four concept blocks: (i) amblyopia, (ii) neurodevelopment/plasticity/network mechanisms, (iii) binocular suppression/imbalance and its quantification, and (iv) treatment modalities and outcomes. An example PubMed query was: (amblyopia OR “lazy eye”) AND (neurodevelopment* OR plasticity OR “critical period” OR “sensitive period” OR cortex OR cortical OR “visual network*” OR neuroimaging OR fMRI OR EEG OR VEP) AND (binocular OR suppression OR “interocular suppression” OR dichoptic OR “contrast balance” OR stereoacuity OR reading OR crowding OR “quality of life”) AND (treat* OR therapy OR patching OR atropine OR “virtual reality” OR “perceptual learning” OR digital). Filters were applied for Humans and English language. Equivalent queries were adapted to Web of Science and Scopus syntax, and reference lists of included reviews and key trials were hand-searched to identify additional eligible studies.

We included peer-reviewed reviews (narrative, state-of-the-art, systematic reviews, and meta-analyses), randomized controlled trials and controlled clinical studies of amblyopia interventions, and key mechanistic human studies providing direct evidence relevant to suppression, sensitive-period plasticity, network-level alterations, and functional phenotypes (e.g., reading-related limitations). We excluded case reports and small uncontrolled series unless uniquely informative for phenotype definition, studies in which amblyopia was not a primary object of analysis, and non-peer-reviewed sources. Papers were screened to identify recent reviews/meta-analyses summarizing areas of agreement and ongoing debate, and primary studies that quantified core constructs and/or reported clinically relevant outcomes. When multiple sources addressed the same point, priority was given to higher-quality designs, clearer outcome definitions, and convergent evidence across independent methods. From each included source we extracted study type, population characteristics (age and etiology when available), mechanistic focus, phenotype metrics (BCVA/logarithm of the minimum angle of resolution (logMAR), stereoacuity when reported, suppression quantification method, reading-related or other functional outcomes, and patient-centered measures), intervention characteristics, and conclusions relevant to mechanistic framing, phenotyping, and predictors/moderators of response. Given the state-of-the-art narrative scope, we did not conduct a preferred reporting items for systematic reviews and meta-analyses (PRISMA)-guided systematic review workflow or a formal risk-of-bias assessment across all included studies. Comparative statements about treatment effects were, therefore, anchored to recent systematic reviews/meta-analyses and major evidence syntheses, and we explicitly noted key design constraints (e.g., outcome heterogeneity and adherence limitations) when interpreting individual trials.

Despite substantial progress, heterogeneity in amblyopia phenotyping and outcome selection—particularly for binocular and functional endpoints—continues to limit cross-study comparability and complicate translation into standardized clinical frameworks [4,28,29,30]. Accordingly, this state-of-the-art review aims to synthesize evidence from 2016–2026 and, on that basis, outline a pragmatic, hypothesis-generating framework for multidimensional phenotyping and mechanism-aligned interpretation of response variability using clinically trackable variables grounded in the reviewed literature [1,4,6,7,28,29,31].

## 3. Historical Evolution and Paradigm Shifts: From Monocular Deficit to Distributed Network Disorder

Amblyopia has historically been treated and reported predominantly as a monocular BCVA deficit [2]. Contemporary syntheses increasingly emphasize that amblyopia reflects atypical binocular experience and experience-dependent plasticity [4,6], and that clinically relevant functional consequences may not be fully captured by BCVA alone [1,6]. This conceptual evolution is clinically relevant because it motivates multidimensional phenotyping beyond BCVA and outcome selection that is informed by the intended mechanism targeted by the intervention (e.g., monocular penalization versus binocular/dichoptic approaches) [1,6]. Table 2 summarizes selected milestones in the conceptual evolution of amblyopia (monocular deficit → sensitive-period plasticity disorder → binocular imbalance → broader multi-domain framing). The table is intentionally illustrative (not exhaustive): entries were chosen to represent review/synthesis anchors within the present reference list and to clarify how different framings emphasize different endpoints and mechanisms, and how broader neurodevelopmental perspectives and developmental constraints can motivate outcome selection beyond acuity in trials and clinical cohorts [1,2,4,6,32,33].

### 3.1. Sensitive Periods and Experience-Dependent Plasticity

Amblyopia is understood as a developmental disorder arising from abnormal visual experience during early life, when experience-dependent changes in visual processing are particularly pronounced [1,2,4]. Experimental and clinical syntheses indicate that plasticity is regulated across development and that sensitive/critical-period constraints contribute to reduced responsiveness and greater constraints on recovery beyond early periods [4]. Mechanistic accounts further describe regulatory processes governing the onset and closure of critical-period plasticity in primary visual cortex (V1) [34]. Developmental analyses also note that infant visual behavior can remain immature despite relatively mature aspects of early neural organization, implying that behavioral development may be constrained by factors beyond early receptive-field properties alone [32]. Collectively, these accounts support a regulated and graded view of plasticity across development rather than a strictly binary ‘on/off’ window [32,34].

### 3.2. Interocular Suppression and Binocular Imbalance

Contemporary syntheses emphasize abnormal binocular interactions in amblyopia, including interocular suppression and binocular imbalance, with implications for phenotyping and rehabilitation [6,23]. Suppression can be quantified using dichoptic paradigms; for example, dichoptic eye-chart approaches estimate suppression using contrast-balance requirements, supporting the use of suppression strength as a graded phenotyping measure rather than a purely categorical label [7]. In deprivation amblyopia due to childhood cataract, interocular suppression can also be quantified; contrast balancing can reduce suppression in many cases at least intermittently, although strong suppression can persist, especially in some delayed-treatment or more severe cases [35]. Together, these findings support considering suppression strength/binocular imbalance as measurable dimensions when interpreting outcomes, especially for binocular/dichoptic interventions [6,7,23,35].

### 3.3. Distributed Cortical Involvement and Stream-Weighted Vulnerabilities

Developmental syntheses indicate that visual behavior can reflect multiple limiting factors beyond early-stage receptive-field maturity, supporting a broader systems-level perspective when interpreting functional consequences in the context of amblyopia [32]. Within this perspective, dorsal-stream vulnerability discussions support considering targeted measurement of motion- and visuomotor-weighted functions in relevant cohorts, without implying a universal or exclusive dorsal deficit across all patients [33]. Functional limitations relevant to reading are supported by clinical synthesis indicating slower reading in amblyopia and the importance of considering the visual and eye-movement demands of reading [8]. Together, these considerations support multidimensional phenotyping beyond BCVA, combining binocular measures (e.g., suppression) and functionally meaningful outcomes (e.g., reading performance), without forcing a single-stream label onto all patients [6,7,8,32,33].

### 3.4. Adult Plasticity: Residual Capacity and Defining Response Across Ages

Critical-period frameworks emphasize developmental constraints on recovery beyond early windows while also recognizing that plasticity is regulated and can be modulated under specific conditions [34]. Evidence syntheses indicate that adults with amblyopia can exhibit measurable improvements with perceptual learning or video game-based training, while emphasizing heterogeneity across interventions, protocols, and outcomes [28]. High-level reviews integrating modern treatment approaches likewise support continued investigation of adult rehabilitation while acknowledging variability in responsiveness and the need for appropriate endpoint selection [29]. Accordingly, in adults, “response” can be operationalized using BCVA/logMAR change together with functionally meaningful outcomes, and—when binocular mechanisms are targeted—a binocular construct such as suppression/binocular balance when feasible [7,28,29]. Outcome heterogeneity is a major barrier to cross-trial comparison and evidence synthesis, particularly when conventional therapies are compared with emerging binocular, dichoptic and digital approaches [27,29]. COSAMS aimed to reduce this by developing stakeholder-informed core outcome sets intended for use in clinical trials and routine practice [31]. For amblyopia, COSAMS reached consensus on ten core outcomes: best corrected visual acuity; near visual acuity; spherical and cylindrical refraction; compliance; treatment-related impact; functionality/long-term impact; ocular alignment; vision-related quality of life; adverse events; and cost [31]. Aligned with COSAMS, we propose a pragmatic multidimensional phenotyping framework designed to remain feasible in clinic-based cohorts while improving mechanistic interpretability and comparability across studies [29,31]. The intent is not to replace BCVA, but to report BCVA within a minimum dataset that also captures binocular function, functional performance, and patient-centred impact—dimensions explicitly represented within COSAMS domains for amblyopia [31].

## 4. Multidimensional Clinical Phenotyping and a Proposed Core Outcome Set (Children and Adults)

A major barrier to translation in amblyopia research and clinical care is inconsistent outcome selection and reporting across studies [29,31]. Consensus initiatives such as COSAMS were developed to harmonize outcome reporting and improve comparability across trials and routine clinical practice, which becomes especially salient when interpreting evidence across conventional therapies (optical correction, patching, atropine) and newer binocular/digital interventions that may engage different therapeutic mechanisms [29,31]. In practice, BCVA remains central, yet COSAMS explicitly incorporates domains beyond acuity—such as compliance and treatment-related and functionality/long-term impacts—supporting a multidomain approach to outcome assessment and reporting in amblyopia [31]. In parallel, contemporary clinical and translational work indicates that clinically meaningful impairment can extend beyond acuity alone, including quantifiable binocular factors (e.g., interocular suppression) and functional limitations such as slow reading and broader functional and health-related quality-of-life impact [8,36,37,38]. Accordingly, amblyopia should be operationalized within a multidomain phenotyping/outcome framework rather than reduced to a single acuity metric, to enable mechanism-aligned interpretation and more informative comparisons across interventions [29,31]. This section outlines a clinically feasible phenotyping framework and a COSAMS-aligned operational minimum dataset/core outcome template intended to support structured interpretation and hypothesis-driven modelling in pediatric and adult settings.

**Figure 1:**
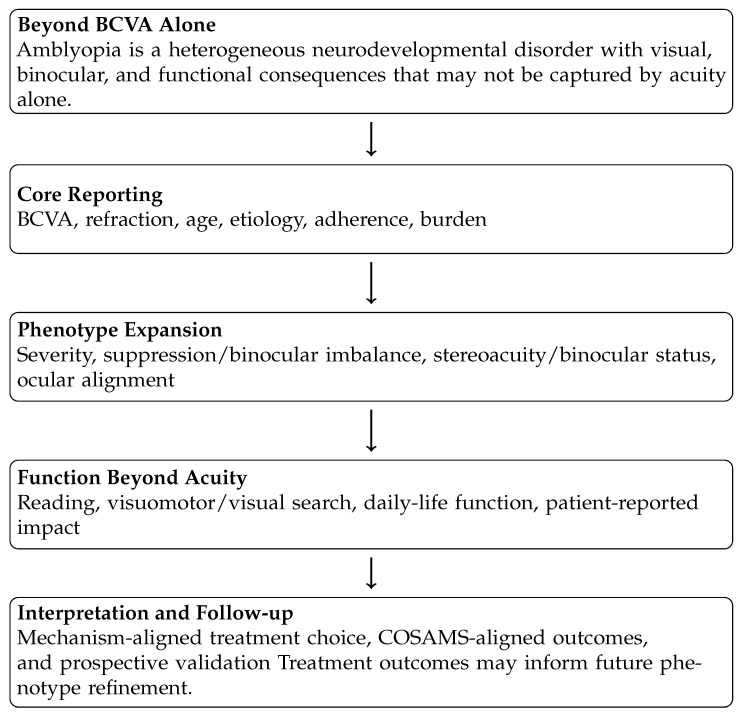
Reader-oriented summary of the multidimensional amblyopia framework, highlighting the central logic of the review: amblyopia should be characterized beyond BCVA alone through core reporting, phenotype expansion, and function-oriented assessment, followed by mechanism-aligned interpretation and standardized outcome evaluation. This conceptual framework is intended to support structured clinical reasoning and future validation rather than serve as a validated predictive tool.

To facilitate navigation of the multidimensional framework developed in this section, Figure 1 provides a simplified reader-oriented summary of the principal assessment and interpretation domains discussed below.

### 4.1. Domain 1: Visual Acuity Is Necessary but Insufficient

BCVA (distance and near) is a core endpoint for amblyopia trials and clinical monitoring and is explicitly included in the COSAMS core outcome set for amblyopia [31]. COSAMS also specifies outcomes beyond acuity—including compliance and treatment-related and functionality/long-term impacts—supporting a multidomain approach rather than reliance on BCVA alone [31]. Therefore, BCVA should be treated as necessary but not sufficient within a COSAMS-aligned multidomain outcome framework [29,31]. Operational recommendation: report best-corrected monocular distance and near visual acuity for both eyes in line with COSAMS; describe the acuity testing approach sufficiently to support interpretability and cross-study comparison [31].

### 4.2. Domain 2: Optical Treatment Response as a Phenotype Marker

Refractive correction alone can produce measurable improvements in BCVA during an initial refractive-adaptation (“optical treatment”) phase in treatment-naïve children with amblyopia, and the duration of this phase is a key design element to report before introducing additional therapy [39]. Because refractive-adaptation duration can influence the child’s visual status at the time penalization begins, inconsistent optical-phase definitions can materially affect interpretation of subsequent treatment effects, particularly in comparative or sequential-treatment designs [39]. Accordingly, optical treatment status (treatment-naïve vs. post-adaptation) and the magnitude of BCVA response during refractive adaptation should be explicitly documented and, when relevant, used as a clinically meaningful stratification/modifier variable in analyses and reporting [39]. Recent randomized evidence further supports the clinical relevance of an initial optical-treatment phase, as the EuPatch trial showed that a meaningful subset of children may achieve clinically meaningful outcomes with refractive correction alone before escalation to patching or atropine strategies [39]. These findings reinforce refractive adaptation as both a therapeutic phase and a clinically informative phenotype marker. Operational recommendation (optical phase): explicitly define and report the refractive-adaptation phase (duration of refractive correction prior to any penalization) and the observed BCVA change over this phase before initiating additional therapy [39]. Recent randomized evidence further supports the clinical relevance of an initial optical-treatment phase, as the EuPatch trial showed that a meaningful subset of children may achieve clinically meaningful outcomes with refractive correction alone before escalation to patching or atropine strategies [39]. These findings reinforce refractive adaptation as both a therapeutic phase and a clinically informative phenotype marker.

#### Graded Optical Penalization as an Intervention-Class Modifier (Bangerter Foils)

Beyond patching and atropine, graded optical penalization using Bangerter foils is used clinically as an alternative fellow-eye image-degradation strategy. Optical/psychophysical studies indicate that Bangerter foils can increase forward light scatter and produce spatial-frequency-dependent degradation of visual performance, with measurable effects on acuity and contrast sensitivity that vary by foil density, supporting their characterization as a controllable image-degradation tool rather than a uniform “occluder” [40,41]. Retrospective clinical data in anisometropic amblyopia further report differences in nonamblyopic-eye ocular growth and refractive trajectories between Bangerter-foil penalization and patching groups, underscoring the importance of clearly specifying the penalization modality and exposure when comparing treatment pathways [42]. Operational recommendation (penalization modality): when penalization is introduced, report the modality (patching/atropine/Bangerter), intended “dose” (e.g., hours/day or foil density), and the verification/adherence approach to improve cross-study interpretability and comparability.

### 4.3. Domain 3: Binocular Function and Quantified Suppression as Core Phenotype Axes

COSAMS defines amblyopia outcomes beyond monocular acuity, including ocular alignment, vision-related quality of life, treatment-related impact, and future functionality/long-term impact, supporting a multidomain characterization of amblyopia burden and treatment effects that is not limited to BCVA alone [31]. Interocular suppression can be operationalized as a graded, quantitative binocular variable in children using dichoptic paradigms [38]. In a pediatric luminance-balance approach, suppression magnitude (interocular luminance difference required for binocular balance) was significantly correlated with interocular visual acuity difference, amblyopic-eye visual acuity, Worth-4-Dot findings, and stereoacuity at both near and far distances, supporting construct validity and positioning suppression strength as a clinically interpretable binocular phenotyping metric rather than a purely qualitative descriptor [38]. However, current evidence for suppression as an independent predictor of treatment response is limited and is largely correlational; prospective validation in structured cohorts is needed to establish prognostic utility [29,31]. Operational recommendation: record binocular function (stereoacuity when measurable, and measurability/status when not). When feasible, include a quantified suppression metric (method specified) and report it alongside COSAMS core outcomes to support structured phenotyping and cross-study interpretability [31,38].

### 4.4. Domain 4: Functional Performance Beyond Acuity

COSAMS includes functionality/long-term impacts within the amblyopia core set, supporting a multidomain approach in which outcomes are not limited to monocular acuity alone [31]. Evidence syntheses further emphasize functional consequences such as reading-related inefficiency/slow reading and broader quality-of-life impact, supporting the inclusion of functional endpoints when the study question concerns daily performance or sustained visual tasks [8,36,37]. Consequently, at least one functional endpoint beyond BCVA is recommended when the clinical question concerns performance in everyday or sustained tasks, and particularly when comparing modalities that may differentially affect function despite similar acuity gains [31]. Operational recommendation: include at least one clearly defined functional endpoint beyond BCVA in structured cohorts/trials when relevant to the study objective, consistent with COSAMS’ emphasis on functionality/long-term impacts [31].

### 4.5. Domain 5: Treatment Burden and Patient-Centred Outcomes

COSAMS includes compliance and treatment-related impacts within the amblyopia core outcome set, supporting the capture of adherence-related and real-world treatment-burden dimensions in trials and clinical reporting [31]. Given that treatment burden and psychosocial effects can influence adherence and patient/family experience, patient-reported outcome measures (PROMs) should be considered as complementary endpoints alongside visual acuity and binocular measures. A systematic review catalogued PROMs used in amblyopia and strabismus and appraised their content coverage and measurement properties, supporting instrument selection aligned with study aims and population [43]. Reviews also synthesize functional and quality-of-life consequences that extend beyond acuity (e.g., reading and health-related quality of life), underscoring the need to quantify patient-centred impact in clinical studies when relevant to the study question [36,37]. In addition, a focused review evaluated psychological distress associated with amblyopia treatment, supporting inclusion of patient- and family-centred outcomes when comparing interventions that differ in burden or adherence demands [44]. Operational recommendation: when feasible, incorporate a validated PROM (instrument specified) chosen using a systematic, psychometric rationale (population, age, language, domains, and measurement properties) rather than defaulting to legacy instruments. For paediatric studies, prioritize instruments that capture treatment burden and family impact; for adult or long-term impact analyses, select vision-specific quality-of-life measures aligned with the study question and validated for the target population (e.g., AmbQoL) [36,43,44,45].

### 4.6. A Pragmatic Minimum Dataset Aligned with COSAMS

To reduce outcome fragmentation and strengthen comparability, we recommend a pragmatic feasibility-focused operational subset aligned with COSAMS for amblyopia: (i) best-corrected distance and near visual acuity, (ii) spherical and cylindrical refraction, (iii) compliance, and (iv) treatment-related and functionality/long-term impacts, with reporting of adverse events (a COSAMS core outcome) and other shared core outcomes where relevant [31]. When feasible, adding a quantified suppression metric (method specified) can further support binocular phenotyping and mechanistic interpretation [38]. Additional binocular function measures (reported with the instrument/metric specified) may further aid stratification in studies where binocular mechanisms are central to the research question, while maintaining COSAMS core outcomes for cross-study comparability [31,38]. This minimum dataset is intended to be implementable in clinical settings while supporting standardized, stakeholder-relevant outcome reporting and comparison across studies [31].

### 4.7. Clinical Phenotypes in Amblyopia: An Integrated Etiology-Based and Function-Based Framework (Children and Adults)

We propose that clinical phenotyping in amblyopia can serve two parallel goals: (i) describe impairment in operational, mechanism-relevant terms (e.g., binocular interaction measures where feasible and functionally relevant limitations), and (ii) inform treatment selection and outcome expectations. BCVA remains the primary outcome measure in conventional amblyopia management, but newer binocular/dichoptic and training-based approaches are explicitly designed to provide different stimulation to the amblyopic and fellow eyes; accordingly, interpretation across modalities may benefit from phenotyping that is not restricted to acuity alone and that can inform mechanism-aligned comparisons [29]. Standardization initiatives such as COSAMS reinforce multidomain outcome reporting beyond monocular acuity—including compliance and treatment-related and functionality/long-term impacts—to enable comparison of results across studies and routine clinical practice [31].

#### 4.7.1. Etiology-Based Phenotypes (Input-Discordance Class)

Amblyopia is commonly classified according to the predominant source of abnormal visual input, including strabismic, refractive, mixed, and deprivation-related forms [1]. Refractive amblyopia includes anisometropic and bilateral ametropic forms. Clinically significant uncorrected astigmatism is also an important cause of abnormal visual development and may be associated with deficits in visual acuity, contrast sensitivity, stereoacuity, and orientation-dependent visual performance, consistent with meridional amblyopia or meridional anisotropies in selected phenotypes [3,46,47]. Mixed amblyopia refers to combined refractive and ocular alignment mechanisms operating in the same patient [1]. We recommend recording etiology explicitly in all structured cohorts and trials as a foundational phenotype descriptor [1]. Etiology is clinically useful for descriptive purposes, and it provides an operational label for the type of early discordant visual experience; accordingly, it may help interpret differences in functional profiles across etiologic groups [1].

Minimal operational variables to record:

- Primary etiology: strabismic/refractive (anisometropic, isoametropic, astigmatic (meridional))/mixed/deprivation-related; specify combined mechanisms when present [1,3].
- Baseline best-corrected distance and near visual acuity for both eyes (report the scale used, e.g., logMAR) [31].
- Baseline interocular acuity difference (IOD), when used as an eligibility or success criterion and/or to support comparability with trials reporting IOD-based endpoints [31,39].
- History of optical correction, including explicit documentation of any optical treatment phase (duration and criteria), because refractive correction alone can produce measurable improvement and can shift visual status before patching in treatment-naïve childhood amblyopia [39].

Clinical implication: In adult intervention syntheses of behavioral therapies, included studies span different training approaches (including monocular and dichoptic protocols) and typically report visual acuity outcomes; therefore, clear etiologic reporting supports transparent sample characterization and stratified interpretation when feasible [1,28].

#### 4.7.2. Function-Based Phenotypes (Expression of the Disorder)

Etiology alone does not fully capture expressed disability; therefore, we propose that a clinically useful phenotype can include function-based dimensions that are mechanistically grounded, measurable, and aligned with the mechanism targeted by the intervention (e.g., monocular penalization versus binocular/dichoptic approaches) [29,31]. We propose four function-based axes that are feasible across children and adults:

Axis A: Acuity phenotype (BCVA severity; interocular acuity difference when reported/used). BCVA remains a core endpoint and is included as a core outcome in COSAMS [31]. Conventional penalization (occlusion and/or pharmacologic penalization) improves BCVA in children; evidence syntheses indicate that atropine penalization is as effective as occlusion in improving visual acuity, and meta-analytic comparisons suggest no statistically significant difference between part-time and full-time occlusion in visual acuity improvement [12,13]. Accordingly, BCVA remains essential for therapy comparisons and longitudinal monitoring [12,13,31].

Axis B: Binocular phenotype (stereoacuity measurable vs. non-measurable; value when measurable). Because binocular/dichoptic approaches explicitly target binocular interactions, documenting binocular status (with the metric specified) can support mechanism-aligned interpretation when feasible [29]. When stereoacuity cannot be measured reliably (e.g., due to age or profound binocular dysfunction), we recommend recording measurability/status explicitly as a phenotype feature rather than treating it as missing data [31].

Axis C: Suppression phenotype (quantified suppression strength when feasible). Suppression can be operationalized as a graded variable in children using dichoptic paradigms [38]. A practical pediatric suppression quantification approach showed significant correlations between suppression strength and multiple clinical measures (including interocular acuity difference and stereoacuity), supporting quantified suppression as a clinically interpretable phenotyping metric and a candidate stratification variable when feasible [38].

Axis D: Functional bottleneck phenotype (reading-limited/task-limited vs. not; quantified when feasible). Functional limitation may include reading-related inefficiency/slow reading and broader everyday impacts not captured by BCVA alone. Disease-impact reviews emphasize effects of amblyopia that extend beyond visual acuity and include impacts on everyday functioning, reading, and health-related quality of life, supporting inclusion of at least one functional endpoint beyond BCVA when the study objective concerns performance in sustained tasks, in both pediatric and adult contexts [8,36,37].

#### 4.7.3. A Pragmatic Phenotype Matrix for Reporting and Hypothesis-Driven Prediction

Combining etiology-based and function-based phenotyping yields a pragmatic matrix usable for clinical documentation, trial reporting, and hypothesis-driven modelling (descriptive, hypothesis-driven; not a predictive algorithm). Example profiles:

- Anisometropic (or mixed with a dominant refractive component), optical responder: this profile highlights the optical treatment phase as a major baseline modifier because refractive correction alone can produce measurable improvement and change visual status before patching [39]. In studies where daily performance is the focus, adding at least one functional endpoint beyond BCVA can help capture impacts that may extend beyond acuity alone [36,37].
- Reduced/absent stereoacuity (including non-measurability) with strong interocular suppression: this profile supports reporting a quantified suppression metric (method specified) alongside acuity measures to support binocular phenotyping and interpretation, particularly when the intervention targets binocular/dichoptic mechanisms [29,38]. Where clinically indicated, conventional occlusion therapy can still improve BCVA, supporting continued use of acuity as a responsive endpoint in longitudinal monitoring and comparisons [12,13]
- Adult amblyopia (any etiology), variable suppression and functional complaints: adult intervention syntheses report heterogeneity/inconsistency across studies and protocols, supporting stratified reporting and cautious cross-study interpretation [28]. When the study question concerns real-world performance, outcome selection can be expanded beyond BCVA to include at least one functional endpoint [36] and, where binocular/dichoptic mechanisms are central, a binocular construct (metric specified) [29].

#### 4.7.4. Patient-Centred Phenotype Modifiers: Burden and Adherence

Two patient-centred modifiers are particularly relevant for clinical translation in pediatric cohorts: treatment burden and adherence. COSAMS includes compliance and treatment-related impact within the amblyopia core outcome set, reinforcing the value of measuring adherence and treatment burden/impact alongside clinical outcomes [31]. Accordingly, validated patient-reported outcome measures (PROMs; instrument specified) can be considered as complementary phenotype modifiers. A systematic review catalogued PROMs used in amblyopia and strabismus and appraised their content coverage and measurement properties, providing an evidence-based basis for instrument selection aligned with study aims and population [43]. Reviews further synthesize functional consequences and health-related quality-of-life impact beyond visual acuity, and a focused systematic review evaluates psychological distress related to amblyopia treatment; together, these support inclusion of patient- and family-centred outcomes when comparing modalities that differ in burden or adherence demands [36,37,44].

### 4.8. Standardized Reporting Template for Amblyopia: A Pragmatic Core Dataset (2026 Proposal)

Heterogeneity in outcome reporting remains a major limitation in amblyopia research and reduces cross-study comparability [31]. COSAMS was developed to reduce this limitation through consensus-based standardization of what should be measured and reported in amblyopia, including best-corrected distance and near visual acuity, spherical and cylindrical refraction, compliance, and treatment-related and functionality/long-term impacts [31]. Standardized outcome adoption is intended to enhance cross-study comparability and evidence synthesis across interventions [31]. Based on the evidence summarized in Section 2 and Section 3, we propose a pragmatic standardized reporting template for clinical trials and structured clinical cohorts in pediatric and adult populations. This template uses COSAMS as a minimum reporting foundation and, when feasible, adds mechanism-oriented variables—such as quantified interocular suppression (method specified) and at least one functional endpoint beyond BCVA—to improve interpretability, particularly when interventions target binocular/dichoptic mechanisms [29,31,38]. Functional endpoints are justified because clinically relevant performance limitations and broader impacts may not be captured by BCVA alone [8,36,37].

Core Reporting Matrix (Minimum Dataset):

COSAMS defines core outcome domains for amblyopia. Table 3 operationalizes a feasibility-focused reporting matrix aligned to COSAMS, including best-corrected distance and near visual acuity, spherical and cylindrical refraction, adherence/compliance, adverse events (when applicable), and patient-relevant treatment impact/functional outcomes [31]. Where binocular/dichoptic mechanisms are targeted, suppression/imbalance should be quantified using a clearly specified method and reported with transparent handling of “not measurable” values [38]. Functional outcomes beyond BCVA are supported by evidence of reading-related inefficiency and broader functional/health-related quality-of-life impact, motivating inclusion of at least one clearly defined functional endpoint when clinically relevant [8,36,37]. Patient-centred burden/impact should be assessed using a validated PROM (instrument specified) informed by PROM evidence syntheses and, when relevant, amblyopia-specific QoL instruments (e.g., AmbQoL) [36,43,44,45]. To improve interpretability across heterogeneous intervention pathways, studies should explicitly report the intervention class and delivered dose/exposure. This applies to established modalities (e.g., optical correction, patching, atropine, and binocular/digital approaches), to graded optical penalization approaches such as Bangerter foils when used [40,41,42], and to selected protocolized orthoptic/vision-therapy programs in phenotype-specific contexts [48,49]. Experimental spectral/filter-based neuromodulation protocols, if studied, should be reported separately as research-context interventions with explicit exposure specification and safety reporting [50,51,52].

Linking the template to hypothesis-driven modelling (brief):

“To support hypothesis-testing cohorts, for example, a pre-specified response variable can be defined as the change in amblyopic-eye best-corrected visual acuity (logMAR) over a defined treatment interval. Within COSAMS-aligned datasets, clinically measurable candidate moderators can then be evaluated as pre-specified hypotheses for explaining variability in visual acuity change, including developmental stage (age), baseline severity, delivered dose/adherence (when measured), and quantified interocular suppression (method specified) when available [29,31,34,38,53]. These hypotheses can be tested using prospectively defined statistical models (e.g., regression or mixed-effects frameworks), with transparent pre-specification of covariate definitions and clinically plausible interactions (e.g., suppression-by-intervention class) where justified by the intervention mechanism. Importantly, this section does not propose a validated predictive equation; it outlines a standardized, testable explanatory modelling strategy facilitated by harmonized phenotyping and outcome reporting. Translational impact: Adoption of this pragmatic template may strengthen cross-study comparability and evidence synthesis by harmonizing core domains without forcing identical study designs [31]. It may also help reduce outcome fragmentation by encouraging pre-specification of essential domains and improve clinical interpretability by aligning outcomes with intervention mechanism and patient-centred impact using validated PROMs (instrument specified) [29,31,36,43,44]”.

## 5. Conceptual Predictive Therapeutic Framework in Amblyopia

### 5.1. Rationale for a Predictive Framework

Despite decades of therapeutic research, amblyopia management in routine practice can remain largely driven by baseline visual acuity and age rather than phenotype-informed. Treatment selection is commonly reported around age and baseline BCVA, yet contemporary syntheses highlight substantial between-patient variability in response and heterogeneity across interventions and endpoints in both pediatric and adult populations [28,29,30,37]. Randomized evidence in children shows that patching improves amblyopic-eye acuity on average, with treatment “dose” being a key design variable, while atropine penalization can be as effective as occlusion in improving visual acuity in the evidence synthesized to date [12,13]. Binocular/digital interventions have also shown measurable short-term amblyopic-eye acuity improvement in some pediatric randomized cohorts [21]. However, residual deficits and variable response profiles indicate that baseline acuity and chronological age alone do not fully account for observed variability, motivating deep phenotyping and mechanism-aligned outcome selection [29,37]. In adults, training-based interventions (perceptual learning and video game-based programs) can yield measurable improvements in pooled analyses, but effects and outcomes vary across studies, and mechanistically relevant moderators are inconsistently reported [28,29]. These patterns motivate a mechanistically grounded framework that integrates multidimensional phenotype variables rather than relying on single endpoints [4,29,37].

### 5.2. Core Conceptual Model

We propose that treatment response in amblyopia is modulated by five interacting domains. The structure is informed by evidence discussed in prior literature and by the synthesis in this review, but requires formal prospective validation before clinical implementation.

#### 5.2.1. Developmental Plasticity Gradient

“Age functions as a probabilistic modifier of neural responsiveness, reflecting residual experience-dependent plasticity rather than a binary sensitive-period threshold [4,34]. Plastic potential is, therefore, best conceptualized as a continuum: higher expected responsiveness in children, with reduced but non-zero residual capacity in adults, consistent with adult rehabilitation evidence summarized in recent syntheses [28,29]”.

#### 5.2.2. Baseline Severity Axis

Baseline BCVA and interocular acuity difference (IOD) define the starting impairment load. Pediatric evidence indicates that meaningful gains can occur under patching-based conventional therapy, with dose considerations influencing outcomes [12,13]. Binocular digital interventions have also shown short-term amblyopic-eye acuity improvement in some pediatric randomized cohorts [21]. However, achievable magnitude may be constrained by baseline severity and measurement ceiling effects, and severity should be interpreted alongside binocular and functional dimensions to avoid over-reliance on acuity-only endpoints [29,36,37].

#### 5.2.3. Binocular Imbalance and Suppression Strength

Interocular suppression is a mechanistically central construct in amblyopia and can be operationalized as a graded variable in children using quantitative dichoptic paradigms [7,38]. A practical pediatric suppression metric demonstrated correlations with interocular acuity difference, amblyopic-eye acuity, Worth-4-Dot findings, and stereoacuity, supporting suppression strength as a candidate stratification variable for structured cohorts and trials [38]. Clinically, greater suppression magnitude is consistent with deeper binocular imbalance and could constrain response to binocularly oriented interventions; however, this moderating role has not yet been prospectively validated across standardized cohorts and should be interpreted as a testable hypothesis rather than an established predictor [29,38]. Conversely, lower suppression may be compatible with more favorable baseline conditions for dichoptic approaches that target binocular interactions [29]. Suppression remains inconsistently quantified across trials, limiting explanatory modelling and cross-study comparability [29,31].

#### 5.2.4. Functional Bottleneck Dimension

Functional limitation in amblyopia is not fully captured by acuity alone. Evidence syntheses emphasize reading-related inefficiency and broader everyday functional and health-related quality-of-life impact, supporting inclusion of at least one functional endpoint beyond BCVA when aligned with the clinical question [8,36,37]. Consequently, it is possible to observe significant BCVA improvement with limited change in functional performance if the dominant bottleneck is not addressed by the intervention mechanism. Outcome interpretation should, therefore, consider whether the phenotype is predominantly acuity-limited or function-limited (sustained-task/reading-related) and include at least one functional endpoint when aligned with COSAMS functionality/long-term impact domains [31].

#### 5.2.5. Treatment Dose and Mechanism Target

Treatment modality interacts with phenotype. Conventional penalization (patching) improves acuity outcomes in children, with treatment dose being a key design variable [13]. Binocular/dichoptic approaches are designed to engage binocular interactions and often aim to reduce interocular imbalance (including suppression) [7,29], while perceptual learning and video game-based approaches in adults have shown pooled acuity improvements across heterogeneous protocols [28,29]. Across modalities, consistent with COSAMS, adherence/compliance and treatment-related and functionality/long-term impacts should be measured and reported; when feasible, patient-centred impact can be captured using a validated PROM (instrument specified) selected using a psychometric rationale aligned with population and domains of interest [31,36,43,44].

### 5.3. Conceptual Interaction Model (Narrative Form)

Treatment response can be conceptualized as the intersection of: plasticity gradient × baseline severity × suppression magnitude × functional bottleneck type × treatment mechanism match × dose/adherence. Rather than a deterministic equation, the model is probabilistic and phenotype-sensitive, and it is intended to be testable within standardized outcome structures (COSAMS, including compliance reporting) and contemporary treatment frameworks [29,31].

### 5.4. Clinical Translation

This framework implies that: (i) two children with similar BCVA may differ in binocular phenotype (e.g., quantified suppression), supporting mechanism-aligned outcome selection and hypothesis-driven stratified analyses rather than assuming identical response profiles [38]; (ii) two adults of similar age may differ in response depending on baseline binocular balance and the match between phenotype dimensions and intervention class, consistent with heterogeneous adult training outcomes reported in syntheses [28,29]; (iii) a function-limited phenotype may require function-targeted outcome selection even after acuity improves [36,37]; and (iv) age alone should not be used to deny therapeutic attempts, because response can be heterogeneous and residual plasticity may remain clinically relevant beyond early childhood in contemporary syntheses [4,29]. The framework is conceptual and does not replace clinical judgment; it organizes it around measurable constructs and mechanism-aligned endpoints, aligned with standardized outcome domains [29,31]. It is informed by evidence discussed in prior literature but requires formal prospective validation before clinical implementation.

### 5.5. Why This Is Necessary in 2026

**Figure 2:**
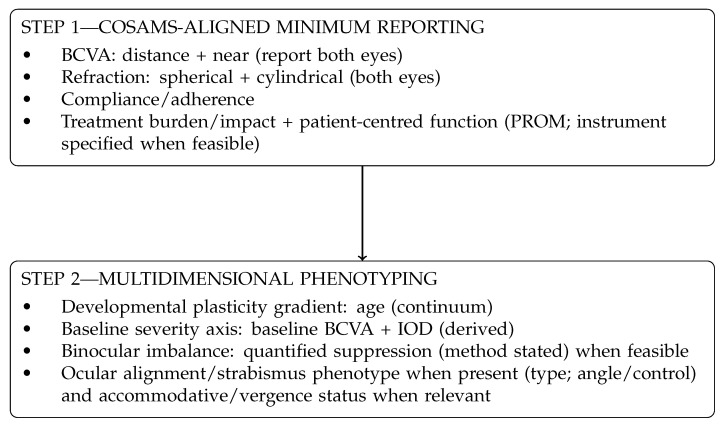

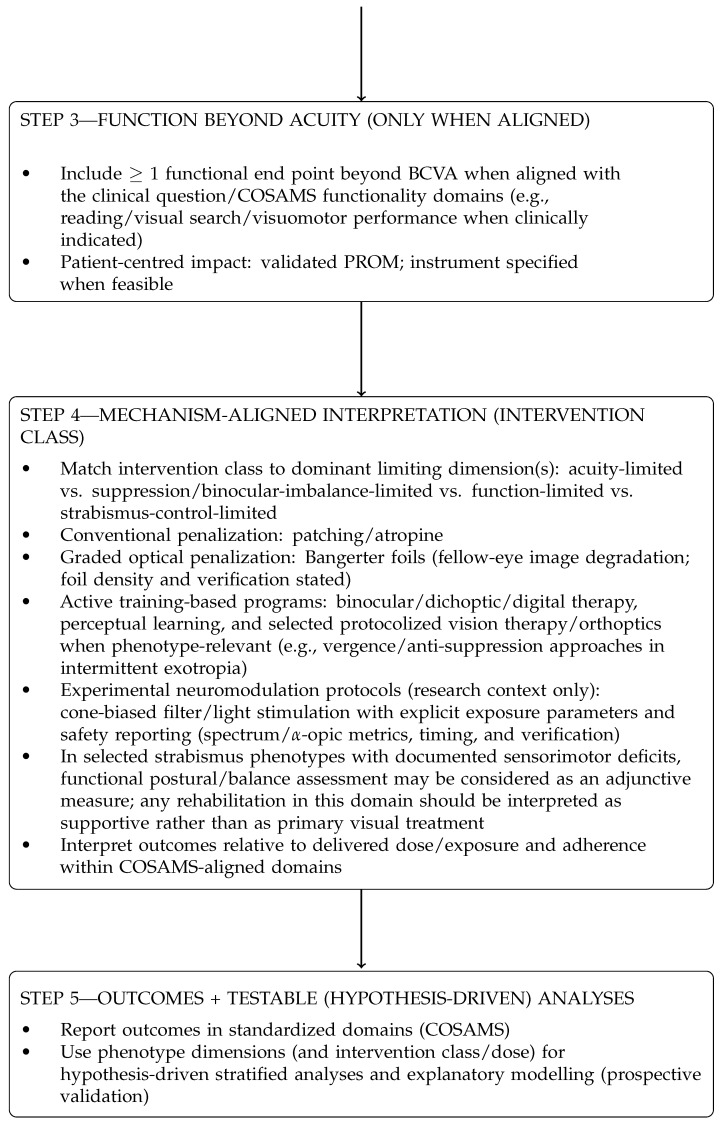
Phenotype-to-treatment route aligned with a conceptual therapeutic framework in amblyopia (not a validated predictive algorithm).

Across contemporary amblyopia research, BCVA remains a dominant endpoint, while quantified suppression, binocular measures, and functional outcomes are less consistently integrated, limiting cross-trial comparability and constraining explanatory modelling [28,29,31]. Without phenotype-integrated reporting aligned to standardized outcome domains (COSAMS), precision-oriented care is difficult to implement and to evaluate consistently across cohorts. This conceptual framework aims to provide a structured bridge between neurodevelopmental mechanisms and translational decision-making and defines testable phenotype dimensions for prospective validation in clinical cohorts [29,31,37]. Figure 2 operationalizes this rationale as a pragmatic phenotype-to-treatment route grounded in COSAMS-aligned minimum reporting [31], with binocular (quantified suppression when feasible) and functional/patient-centred measures incorporated when aligned with the clinical question, to support standardized, hypothesis-driven evaluation in prospective cohorts [28,29,31] rather than a validated predictive algorithm.

This schematic maps the Section 4 framework into a pragmatic route: COSAMS-aligned minimum reporting, multidimensional phenotyping (developmental stage, baseline severity, quantified suppression when feasible), addition of at least one functional endpoint beyond BCVA when aligned with the clinical question, and mechanism-aligned interpretation across intervention classes [31]. Conventional penalization improves amblyopic-eye acuity on average in children, with patching dose as a key design variable, and atropine penalization can be as effective as occlusion in synthesized evidence [12,13]. Binocular/dichoptic approaches are designed to engage binocular interactions, and baseline suppression/imbalance may be a relevant stratification dimension when suppression is quantified [7,29,38]. In adults, training-based approaches show pooled acuity improvements across heterogeneous protocols and outcomes [28,29]. In addition to conventional penalization and binocular/dichoptic or other training-based approaches, the broader treatment landscape may include graded optical penalization (e.g., Bangerter foils) [40,41,42] and selected protocolized orthoptic/vision-therapy programs in phenotype-specific contexts such as intermittent exotropia [48,49]. Experimental cone-biased spectral neuromodulation should be interpreted separately as a research-direction framework requiring prospective evaluation, explicit exposure specification, and safety reporting before any broader clinical positioning [50,51,52]. The framework is conceptual, intended for hypothesis-driven testing within standardized outcomes (COSAMS), and requires prospective validation [29,31].

Note: Conceptual framework. Not a validated predictive algorithm. Requires prospective validation.

## 6. Neurofunctional and Multisensory Consequences of Amblyopia/Strabismus—Why BCVA-Only Endpoints Are Insufficient

Evidence from 2016–2026 supports that amblyopia and strabismus can be associated with functional impacts beyond monocular acuity, extending to fine visuomotor control, visuospatial/perceptual processing, oculomotor–attention coupling, and—in selected strabismus phenotypes—cross-modal attentional findings. These broader consequences align with syntheses emphasizing impacts beyond acuity and can remain clinically relevant even when acuity improves, motivating mechanism- and question-aligned outcome selection beyond BCVA when functional participation is impacted [37,54,55,56].

### 6.1. Visuomotor Performance and Motor Competence

A systematic review and meta-analysis focusing on fine visuomotor performance reported poorer fine visuomotor skill performance in amblyopia compared with typical-vision peers, while also emphasizing substantial heterogeneity in tasks and outcome definitions that constrains synthesis and clinical translation [54]. Complementary evidence from population-based data indicates an association between amblyopia status and reduced motor competence in school-age children, supporting potential downstream impacts on daily activities and participation [55]. Experimental task paradigms further show altered eye-hand coordination during visually guided reaching in children with monocular-deprivation amblyopia, consistent with sensorimotor integration constraints beyond letter recognition [57]. In adults, functional impacts spanning fine motor skills, reading speed, and self-reported quality of life have also been reported in amblyopia and/or strabismus cohorts, reinforcing that functional consequences may persist across the lifespan [11].

### 6.2. Visuoperceptual and Visuomotor Integration Profiles in Clinical Instruments

Clinical profiling using standardized instruments provides a pragmatic route to quantify visuoperceptual and visuomotor integration differences relevant to learning and daily functioning [58,59]. In a prospective pediatric cohort with strabismus (with and without amblyopia), TVPS-3 and Beery VMI-6 performance varied in relation to strabismus characteristics/binocularity state/visual acuity (as analyzed in that cohort), supporting multidimensional phenotyping beyond “acuity-only” definitions [59]. In contrast, a multicenter retrospective study reported no systematic TVPS differences between strabismus/amblyopia, binocular/accommodative dysfunction, and control groups, but observed high DEM variability and suggested associations between horizontal DEM time and exotropia magnitude, underscoring that oculomotor and perceptual outcomes can show clinically meaningful dispersion even when group means are similar [58]. “Complementing instrument-based clinical profiling, recent evidence suggests that children with anisometropic amblyopia may also show alterations in visual cognitive functions, supporting the view that functional consequences can extend beyond visual acuity loss alone [60]”.

Together, these mixed findings support reporting both central tendency and dispersion (e.g., variance/percentiles) and, where feasible, phenotype-stratified analyses rather than sole reliance on group averages [58,59].

### 6.3. Attention, Visual Search, and Sustained-Task Constraints

Beyond paper-and-pencil measures, experimental paradigms have reported measurable differences in visual attention and visual search in children with amblyopia, implicating attentional selection and scanning efficiency as potential functional bottlenecks for tasks requiring sustained, efficient exploration (e.g., classroom scanning and reading-related behaviors) [10]. Complementary evidence indicates that children with amblyopia may make more saccadic fixations during visual search tasks, supporting a measurable alteration in visual search behavior at the oculomotor level [61]. A recent systematic review and meta-analysis further supports increased saccadic latency in amblyopia (largest under amblyopic-eye viewing, with smaller but significant effects under binocular viewing), reinforcing the clinical relevance of oculomotor-attention efficiency beyond acuity alone [62]. In adults, psychophysical work has reported face-perception deficits implicating both bottom-up and top-down constraints, supporting persistence of higher-order functional limitations beyond acuity in some cohorts [63].

### 6.4. Multisensory and Cross-Modal Considerations in Strabismus Phenotypes

As an adjacent, phenotype-specific extension beyond core amblyopia outcomes, multisensory considerations may be relevant in selected strabismus phenotypes. In children with intermittent exotropia, objective testing has identified combined visual and auditory attention deficits, suggesting that attentional network alterations may extend beyond purely visual processing domains in this phenotype [56]. These findings support considering broader attentional domains when clinically indicated, while recognizing that multisensory effects are phenotype- and task-dependent. Consistent with multisensory integration demands, a 2021 systematic review and meta-analysis reported that children with strabismus exhibit poorer postural control than age-matched controls, with significantly altered center-of-pressure measures under stable standing conditions. The findings are consistent with the concept that abnormal visual input in strabismus may disturb multisensory integration for balance, potentially increasing reliance on vestibular and somatosensory cues. Although study heterogeneity was high and the review does not test rehabilitation efficacy directly, it supports consideration of postural and multisensory assessment within broader functional evaluation of pediatric strabismus and, where clearly indicated by documented deficits, may justify exploratory adjunctive balance-oriented rehabilitation as a supportive rather than primary visual-treatment component [64].

### 6.5. Neuroimaging Correlates: Distributed-Network Perspective with Current Limits

Converging neuroimaging studies in pediatric strabismus with amblyopia report altered interhemispheric homotopic connectivity and disrupted functional connectivity within visual and attention-related networks relative to controls [65,66]. Task-based fMRI evidence also suggests altered motion-salience attention processing accompanied by reduced functional connectivity between frontal eye fields and visual cortex in strabismic amblyopia, consistent with a distributed-network account beyond a purely acuity-based framing [67]. In adults with intermittent exotropia, resting-state connectivity alterations across visual, dorsal attention, executive-control, and auditory-related networks have been reported, with associations to clinical characteristics [68]. However, across these imaging literatures, the evidence base remains predominantly cross-sectional and correlational; longitudinal replication and prospective linkage to treatment outcomes are required before proposing these measures as treatment-response biomarkers or predictive clinical tools [65,66,67,68,69].

#### Clinical Implication

Collectively, these strands support expanding outcomes beyond BCVA alone when the clinical question warrants it. A pragmatic, tiered reporting approach is to include: (i) core vision endpoints (BCVA, stereoacuity, and suppression quantification when feasible); (ii) oculomotor function and reading/visual-search measures when learning complaints or sustained-task difficulties are present; and (iii) visuomotor and attention measures when functional participation is impacted (e.g., handwriting, sports, balance, and classroom scanning). This tiered approach is consistent with COSAMS-aligned, patient-relevant outcome selection and contemporary syntheses emphasizing impact beyond acuity [10,31,36,37,44,54,61]. These clinical considerations also highlight key limitations of the current evidence base, summarized in the next section.

## 7. Limitations of the Current Evidence Base (And Implications for Interpretation)

Because functional outcomes and binocular measures are increasingly clinically relevant, limitations in outcome selection, method-dependent suppression quantification, and long-term follow-up (including patient-centered outcomes) constrain cross-study comparability and translation to clinical decision-making.

### 7.1. Heterogeneity of Outcome Measures

A persistent limitation in amblyopia research is heterogeneity of outcome definitions and reporting across conventional penalization, binocular/dichoptic, and training-based approaches, which constrains synthesis and mechanistic interpretation [4,28,29]. Although COSAMS was developed to reduce this fragmentation, multidomain outcomes beyond BCVA are still not reported consistently across studies [31]. Even though BCVA is commonly reported, binocular metrics (e.g., stereoacuity) and quantified suppression, as well as functional outcomes beyond acuity (e.g., reading-limited performance), are not captured or reported uniformly across studies, reducing mechanistic interpretability and complicating cross-study comparison [4,8,29,31]. In pediatric randomized trials of conventional therapy, primary endpoints are often acuity-based (BCVA change), establishing efficacy on average but limiting phenotype-driven interpretation when binocular or functional modifiers are not concurrently captured [13,21,29]. In adult evidence syntheses of perceptual learning and video game-based programs, pooled effects are measurable but heterogeneity across protocols, dose, and outcomes remains substantial, and moderator reporting is inconsistent—again limiting inference across cohorts [28,29].

Clinical implication: without standardized multidimensional reporting aligned to core outcome domains, apparent variability in effect sizes may be influenced, at least in part, by between-cohort differences (phenotype composition) and endpoint selection, in addition to any true differences in treatment efficacy across mechanisms [4,28,29,31].

### 7.2. Incomplete Suppression Reporting and Method Dependence

Suppression and binocular imbalance are widely treated as core mechanistic constructs in amblyopia, with direct implications for phenotype definition, endpoint selection, and the interpretation of binocularly oriented therapies [4,6,29]. Importantly, suppression is not only a qualitative descriptor: it can be quantified using dichoptic paradigms, supporting its use as a graded phenotyping variable [7]. In pediatric populations, suppression metrics have demonstrated clinically relevant correlations with interocular acuity difference, amblyopic-eye acuity, Worth-4-Dot findings, and stereoacuity, supporting suppression strength as a candidate stratification dimension for structured cohorts and trials [38]. Despite this mechanistic relevance and available quantitative methods, suppression is not quantified or reported uniformly across trials. Moreover, when suppression is assessed, method variability can limit cross-study comparability, thereby hindering phenotype-driven explanatory modelling and mechanistic synthesis [29,31]. Clinical implication: incomplete suppression reporting undermines the ability to (i) stratify patients mechanistically, (ii) interpret binocular-targeting interventions using mechanism-aligned endpoints, and (iii) compare treatment effects across cohorts when baseline binocular imbalance differs [6,7,29,31,38]. Importantly, many neuroimaging findings in amblyopia and strabismus remain cross-sectional and cohort-dependent; replication across independent samples and prospective linkage to treatment outcomes are required before translation into predictive clinical tools [69].

### 7.3. Limited Long-Term Functional Follow-Up and Patient-Centred Outcomes

Compared with the short-term BCVA-focused trial literature, long-term reporting that links acuity gains to sustained functional and patient-perceived benefit is less frequently available, limiting confidence about real-world durability and impact [31,36,37,43,44]. Recent integrative reviews highlight that amblyopia can have lasting consequences beyond acuity, affecting reading, visuomotor skills, participation, and health-related quality of life—motivating systematic capture of functional and patient-centred outcomes in both pediatric and adult cohorts [36,37]. COSAMS explicitly includes domains beyond monocular acuity, including functionality/long-term impacts and compliance/treatment-related impacts, reinforcing that clinically meaningful benefit may extend beyond BCVA change alone [31]. PROM selection should follow a systematic, psychometric rationale (population, domains, and measurement properties), as summarized in a PROM-focused systematic review for amblyopia and strabismus [43]. In addition, treatment burden and psychological distress may influence delivered dose and continuation, further supporting patient- and family-centred outcomes in trials and clinical audits [44]. Translational implication: when studies emphasize BCVA alone and omit functional and patient-centred domains, a gap remains between short-term visual improvement measured in clinic and real-world benefit as experienced over time [31,36,37,43,44].

### 7.4. Pediatric–Adult Comparability Constraints

Although evidence syntheses support measurable improvements in adults under structured training paradigms [28,29], direct comparability between pediatric RCTs and adult intervention evidence is limited because of differences in baseline phenotype composition and study design. Adult cohorts can differ from pediatric trial samples in treatment history, baseline severity distribution, and endpoint selection, and adult evidence shows substantial heterogeneity across protocols and outcomes [28,29]. In parallel, pediatric randomized trials of conventional therapy establish average acuity gains in defined age ranges but may not routinely capture the same constellation of mechanistic and functional modifiers emphasized in adult training and binocular intervention literature [13,21,29]. Interpretive implication: the comparison between pediatric trial outcomes and adult intervention outcomes should be interpreted with caution unless phenotype variables (etiology, baseline severity, binocular imbalance/suppression, and functional bottlenecks) are explicitly accounted for and endpoints are aligned within standardized outcome structures [4,28,29,31].

### 7.5. Lack of Predictive Stratification and Prospective Validation

Despite major progress in available therapies, amblyopia management and many trial designs do not consistently incorporate explicit phenotype-based stratification based on quantified suppression or treatment-mechanism matching, even though contemporary syntheses emphasize these constructs as central to understanding response variability [4,28,29]. COSAMS provides a pragmatic foundation for standardization and comparability, but prospective validation studies integrating multidimensional phenotype data—including binocular/suppression quantification and functional endpoints when clinically relevant—are not yet widely available, constraining the development of robust prognostic and explanatory models [29,31]. Research implication: this limitation is also an opportunity. Hypothesis-testing cohorts that implement COSAMS core outcomes alongside mechanism-relevant phenotype axes (suppression/binocular measures and functional endpoints) are positioned to test moderators of response and advance precision-oriented amblyopia care [29,31,38].

## 8. Research Agenda 2026–2030

### 8.1. Standardization of Core Outcomes

Given the persistent heterogeneity outlined above, future trials and structured cohorts should prioritize a minimal shared reporting set aligned with COSAMS to improve comparability and mechanistic interpretability across intervention classes [4,28,29,31]. Accordingly, future trials and structured cohorts should adopt a minimal standardized set that supports phenotype-aware interpretation, including:

- Monocular BCVA (distance and, when relevant, near), preferably reported in logMAR to support comparability and enable interocular difference (IOD) computation [31].
- Stereoacuity, reported quantitatively when measurable, with ‘not measurable’ explicitly recorded when applicable [31].
- A clearly specified, quantified suppression metric (method, units, and testing conditions stated), given the availability of quantitative paradigms and the relevance of suppression as a graded phenotyping variable [7,38].
- At least one functional endpoint beyond acuity when aligned with the clinical question (e.g., reading-related limitation), consistent with evidence that amblyopia can impact reading-related performance and with COSAMS inclusion of functionality/long-term impacts [8,31].
- Treatment adherence/compliance reporting (and, when feasible, delivered dose), aligned with COSAMS emphasis on compliance and treatment-related impacts [31].
- A patient-centred burden/impact measure to interpret real-world effectiveness and persistence of impact, selected based on PROM synthesis and evidence on functional/QoL consequences and psychological impact (instrument specified) [36,43,44].

### 8.2. Phenotype-Stratified Trial Design

“Contemporary syntheses consistently emphasize substantial between-patient variability in amblyopia treatment response and highlight binocular imbalance and functional limitations as mechanistically relevant dimensions that are not fully captured by BCVA alone [4,29].” To improve interpretability, future studies should incorporate phenotype-aware design features, either through stratified enrollment or prespecified subgroup analyses, based on measurable baseline constructs.

Where feasible, studies should prespecify stratification (or subgroup analyses) by:

- Etiology (anisometropic, isoametropic, astigmatic (meridional), strabismic, deprivation, mixed), because etiology is clinically meaningful and may influence phenotype composition and interpretation [1,3,29].
- Baseline suppression magnitude, quantified with a stated method, because suppression can be operationalized in children and has demonstrated clinically relevant associations with standard binocular and clinical findings [7,38].
- Functional phenotype when relevant (e.g., acuity-dominant vs. reading-related limitation; and/or oculomotor/visual-search-attention constraints), consistent with evidence that amblyopia can be associated with slower reading and altered visual search behavior [8,61].
- Age as a continuous moderator rather than a binary cut-off, consistent with contemporary syntheses emphasizing heterogeneous response across age ranges [4,28,29]. This approach structures heterogeneity rather than ignoring it, facilitating mechanism-aligned interpretation within standardized outcome reporting [29,31].

### 8.3. Prospective Validation of Phenotype-Driven Frameworks

The conceptual phenotype-driven framework proposed in Section 4 should be evaluated prospectively using standardized baseline phenotyping and prespecified endpoints, because heterogeneity in protocols and outcomes limits post hoc inference and cross-study comparability [28,29,31]. Key design features include:

- Multicenter structured datasets with shared measurement definitions and reporting templates aligned to COSAMS domains [31].
- Baseline phenotyping that includes severity (BCVA/IOD), etiology, and binocular status (e.g., stereoacuity where applicable and quantified suppression with method and units stated), plus at least one functional endpoint beyond acuity when aligned to the research question (e.g., reading-related limitation) [7,8,29,31,38].
- Prespecified responder definitions using clinically interpretable thresholds and, when appropriate, parallel responder definitions for monocular, binocular, and functional outcomes to match the intended mechanism of the intervention [29,31].
- Inclusion of both pediatric and adult cohorts, treating age as a continuous moderator and maintaining mechanism-aligned endpoint selection in the presence of heterogeneity in adult training outcomes [4,28,29].

### 8.4. Biological Measures as a Future Direction (Feasibility First)

Contemporary reviews increasingly frame amblyopia as a neurodevelopmental disorder involving binocular interactions and distributed visual processing rather than a purely monocular acuity deficit [4,6,29]. On this basis, biological measures may be explored to refine mechanistic phenotyping and to help contextualize response variability, but for clinical scalability, any added measures should be evaluated against feasibility, reproducibility, and incremental value beyond standardized clinical phenotyping and core outcome reporting [29,31]. Biological measures should, therefore, be positioned as adjunctive and hypothesis-driven rather than as replacements for clinically accessible phenotype axes [29,31]. In parallel, neuroimaging findings in strabismus-amblyopia and related phenotypes support a distributed-network perspective (i.e., network-level differences beyond acuity), but the current evidence base remains largely cross-sectional and correlational; therefore, these measures should be interpreted as candidate neural correlates requiring longitudinal replication and prospective linkage to treatment outcomes before any treatment-response predictive clinical role is claimed [65,66,67,68]. A pragmatic feasibility-first roadmap can be structured as a mixed A–B–C axis approach, in which additional measures are judged by reproducibility, scalability, and incremental value beyond COSAMS-aligned clinical phenotyping and core outcome reporting [29,31].

#### 8.4.1. Axis A (Structure): Feasibility-Anchored Structural Markers

Structural MRI-derived markers may support hypothesis-driven mapping of structural differences across visual and related systems. Recent MRI literature in anisometropic and strabismic amblyopia includes structural and microstructural approaches (including diffusion-based techniques) that report brain-level differences across cohorts, supporting the rationale for Axis A as a mechanistic layer rather than a routine clinical requirement [69,70]. Within a feasibility-first framework, Axis A should be considered only when acquisition, preprocessing, and reporting are clearly standardized for the selected marker and cohort, and when it is expected to add value beyond COSAMS-aligned clinical phenotyping and core outcome reporting [29,31].

#### 8.4.2. Axis B (Functional Connectivity/Network Organization): Distributed-Network Correlates from rs-fMRI and Task-fMRI

Resting-state fMRI studies in pediatric strabismus-amblyopia cohorts report altered interhemispheric functional connectivity and resting-state connectivity organization measures consistent with network-level differences in visual and attention-related systems [65,66]. In task-based fMRI, strabismic amblyopia has been associated with impaired activation of motion-salience attention processing accompanied by reduced functional connectivity between frontal eye fields and visual cortex, supporting a distributed-network phenotype beyond a purely acuity-based characterization [67]. Findings in adult intermittent exotropia also indicate altered resting-state network connectivity with reported associations with clinical characteristics, reinforcing the relevance of network-level characterization across strabismus phenotypes [68]. Collectively, these results are informative for mechanistic framing, but do not yet constitute validated biomarkers for treatment-response prediction; therefore, Axis B measures should be interpreted as candidate neural correlates requiring longitudinal replication and prospective linkage to treatment outcomes [65,66,67,68].

#### 8.4.3. Axis C (Quantitative Brain Activity): Scalable Electrophysiology as a Translational Route

Scalable electrophysiological measures can provide quantitative indices of binocular interaction and visual-cortical processing. Cortical electrophysiology paradigms have been used to estimate interocular interaction components consistent with excitatory and suppressive contributions in strabismic amblyopia, supporting an interpretable mechanistic readout at the cortical level [71]. Systems-level recordings (e.g., MEG) further report reduced evoked activity and altered cortical oscillations in anisometropic amblyopia, with associations to visual acuity impairment, reinforcing the relevance of quantitative brain-activity endpoints for mechanistic characterization [72]. Clinically oriented pattern-VEP studies also report amplitude/latency differences and associations with foveal sensitivity in amblyopia, often with overlap between groups—supporting use as an adjunctive mechanistic endpoint rather than a stand-alone discriminator [73]. In addition to conventional clinical visual evoked potential (VEP) protocols, specialized electrophysiological paradigms may provide enhanced sensitivity for selected amblyopia phenotypes. For example, orientation-specific pattern-onset VEP recordings have demonstrated abnormal neural responses in children with bilateral refractive amblyopia compared with non-amblyopic controls, supporting the value of phenotype-targeted electrophysiological assessment beyond standard clinical protocols [74]. Within a feasibility-first framework, Axis C should be introduced only with prespecified protocols and endpoints, and after demonstrating incremental value beyond core clinical phenotype variables and COSAMS-aligned outcome reporting [29,31]. Accordingly, quantitative brain activity measures should be positioned as adjunctive and hypothesis-driven layers rather than replacements for clinically accessible phenotype axes [29,31].

Overall, a feasibility-first A–B–C framework supports hypothesis-driven mechanistic layering, while underscoring that current biological evidence is not yet sufficient to justify routine clinical use as treatment-response predictive tools [29,31]. To maximize comparability, protocols should prespecify measurement methods and reporting templates and ensure transparent reporting of missingness and ‘not measurable’ values within COSAMS-aligned domains [31]. Across axes, the priority remains rigorous COSAMS-aligned clinical phenotyping and core outcome reporting; biological measures should be added only when they demonstrably improve interpretability beyond this baseline and can be implemented reproducibly at scale [29,31]. Aligned with this feasibility-first logic, two additional translational directions—protocol-standardized spectral (cone-biased) neuromodulation and protocolized vision-therapy/orthoptics programs in selected phenotypes—can be positioned as research-ready intervention pathways for prospective evaluation, provided exposure/dose parameters are explicitly specified and outcomes are assessed within COSAMS-aligned domains.

### 8.5. Cone-Specific Filter-Based Neuromodulation: Protocol Standardization as a Trial-Ready Research Direction

An emerging methodological direction is the standardization of protocol reporting for spectrally selective (cone-biased) light/filter stimulation studied as an experimental neuromodulation framework in amblyopia and strabismus. A recent Clin Pract framework consolidates cone-biased filter classes and integrates α-opic metrology with photobiological and temporal-modulation safety standards to define reproducible reporting parameters, explicitly positioning these specifications as methodological anchors for prospective evaluation rather than as evidence of clinical efficacy [50]. Earlier studies in strabismus/amblyopia cohorts reported measurable electrophysiological changes and concurrent changes in visual-performance measures across a structured cycle of light-based stimulation, supporting feasibility and motivating prospective, controlled trials designed to quantify clinical benefit using COSAMS-aligned outcomes (BCVA, binocular measures, and function when relevant), with transparent exposure specification and safety reporting [51,52].

### 8.6. Protocolized Visual Therapy Programs for Binocular Dysfunction in Selected Phenotypes

Evidence for protocolized vision therapy (VT) or orthoptic programs is emerging in selected pediatric binocular-disorder phenotypes, but effects appear phenotype-, protocol-, and endpoint-dependent and should not be generalized across amblyopia or strabismus populations. In amblyopia, a randomized trial comparing patching alone versus patching combined with a structured VT protocol reported greater visual acuity gains with the combined regimen under the tested protocol [9]. In strabismus, randomized evidence in children with small-to-moderate angle intermittent exotropia indicates that office-based vergence and anti-suppression therapy (with home reinforcement) can improve exotropia control and binocular outcomes versus observation over the short term, supporting VT/orthoptics as a non-surgical option in selected intermittent exotropia phenotypes under protocolized delivery and measured adherence [48,49]. Taken together, these approaches are best viewed as phenotype-sensitive investigational directions that require prospective evaluation with explicit dose/exposure specification, adherence tracking, and COSAMS-aligned outcome reporting before any broader clinical positioning.

## 9. Conclusions

By 2026, amblyopia is increasingly understood as a multidimensional neurodevelopmental disorder of vision in which binocular imbalance, atypical visual processing, and clinically relevant functional limitations are not fully captured by BCVA alone [4,6,29]. Although established therapies can produce clinically meaningful improvements, treatment response remains heterogeneous across ages, etiologies, protocols, and outcome definitions in both pediatric and adult populations [13,28,29,36,37]. Recent evidence, including the EuPatch trial, further highlights the importance of explicitly documenting the optical-treatment phase, since refractive adaptation may itself produce meaningful gains and influence interpretation of subsequent treatment effects [39]. Taken together, current evidence supports moving beyond age- and acuity-only decision-making toward multidimensional phenotyping that incorporates etiologic stratification, binocular imbalance and suppression when feasible, and functionally relevant constraints when clinically indicated [7,8,29,31,38]. Key priorities for the next phase of the field include COSAMS-aligned standardized reporting, transparent documentation of adherence and treatment burden, and prospective validation of phenotype-driven frameworks across intervention classes. Emerging and phenotype-specific approaches should be evaluated in prospective studies with transparent reporting and standardized outcomes before broader clinical implementation [29,31,36,40,41,42,43,44,45,48,49,50,51,52].

## Figures and Tables

**Table 1:** Representative evidence for binocular/dichoptic and digital amblyopia therapies (selected randomized controlled trials (RCT) + synthesis-level evidence; pediatric unless noted).

Study/Evidence	Population	Intervention Class	Comparator	Outcomes Emphasized (Per Study)	What It Contributes to Section 1
Kelly et al., 2016 (RCT) [14]	Children	Binocular iPad game (dichoptic game)	Patching	BCVA (primary at 2 weeks, crossover design) + stereoacuity/suppression outcomes (secondary); adherence/engagement	Early crossover RCT for binocular iPad paradigms; illustrates short-term BCVA change and the importance of adherence/engagement
Holmes et al./ PEDIG, 2016 (multicenter noninferiority RCT) [15]	Children 5–12 y	Binocular iPad game (falling-blocks-type dichoptic game; not Dig Rush)	Part-time patching	BCVA (primary) + binocular outcomes; adherence; trial design (head-to-head RCT design)	Landmark multicenter head-to-head RCT vs. patching; highlights adherence and endpoint/design dependence in interpretation
Manny et al., 2022 (RCT) [21]	Children 4–6 y	Binocular Dig Rush game + spectacles	Spectacles alone (continued optical correction)	BCVA (primary at 4 weeks; follow-up at 8 weeks) + binocular/stereo outcomes; adherence	Adds younger-cohort RCT with optical-only comparator; illustrates time-point dependence in interpretation (greater improvement at 4 weeks; between-group differences less clear by 8 weeks)
Xiao et al., 2022 (Phase 3 RCT) [20]	Children 4–7 y	Dichoptic digital therapeutic (home) + spectacles	Glasses-only (per protocol)	BCVA (primary at 12 weeks) + safety/adherence	Phase 3 “home-based digital therapeutic” RCT with optical-only control arm
Wygnanski-Jaffe et al., 2023 (multicenter RCT; CureSight pivotal) [16]	Children	Eye-tracking-based dichoptic home treatment	Patching	BCVA (primary); + stereo/binocular outcomes; adherence	Key home-based platform RCT vs. patching; emphasizes trial design and adherence as interpretation drivers
Wygnanski-Jaffe et al., 2025 (RCT; high-adherence CureSight) [17]	Children	Eye-tracking-based dichoptic home treatment (high-adherence conditions)	Patching	BCVA + adherence comparison; binocular/stereo outcomes (as reported)	Adds adherence-focused evidence within the same platform class; supports adherence as a moderator of observed effect.
Jost et al., 2022 (RCT; streaming dichoptic movies) [19]	Children 3–7 y	Streaming dichoptic movies (home)	Patching	BCVA (primary at 2 weeks) + feasibility/adherence; binocular outcomes (as reported)	Home-streaming approach; illustrates feasibility and primary time-window dependence in interpretation.
Jost et al., 2024 (Pilot RCT; streaming cartoons) [18]	Children 3–5 y	Contrast-rebalanced dichoptic cartoons (home; pilot)	Patching	BCVA + feasibility; binocular/suppression measures (as reported)	Extends evidence to very young children; pilot design and inclusion of binocular/suppression measures where reported.
Meqdad et al., 2024 (RCT; mixed ages) [22]	Ages 6–37 (includes adults)	VR-based dichoptic stimulation	Patching	BCVA (post-treatment and follow-up) + binocular/stereo outcomes (as reported)	RCT example including mixed-age participants; interpretation depends on protocol, endpoints, and age composition.
AAO Report (evidence synthesis) [23]	Children/ teens (evidence base)	Binocular treatments (class-level)	Conventional therapies across studies	Summary of RCT evidence; limitations (incl. adherence/outcome heterogeneity)	High-authority synthesis supporting “mixed evidence + heterogeneity; superiority not established” framing.
Meta-analysis of RCTs (binocular vs. patching) [24]	Pediatric RCTs	Binocular therapies (class-level)	Patching/ controls	BCVA + stereo/binocular endpoints; between-study heterogeneity	Pooled estimate across RCTs; supports “small/inconsistent between-group differences” and heterogeneity emphasis.
Meta-analysis of RCTs (binocular vs. patching) [25]	Pediatric RCTs	Binocular therapies (class-level)	Patching	BCVA outcomes; between-study heterogeneity	Reinforces that conclusions depend on trial selection and heterogeneity; not a uniform advantage over patching.
Systematic review + meta-analysis (children/teens) [26]	Children/ teens	Vision-based treatments (includes binocular approaches)	Conventional controls across trials	BCVA + binocular outcomes; heterogeneity drivers	Broad evidence map consistent with “endpoint/adherence/heterogeneity-driven interpretation” narrative.

Note: Selected trials; dosing and timepoints are protocol-specific; outcomes listed reflect endpoints reported in each publication; table is illustrative (non-exhaustive).

**Table 2:** Selected milestones in the conceptual evolution of amblyopia (illustrative).

Era/Milestone Framing	Dominant Framing	Primary Clinical Endpoint(s) Commonly Emphasized	Mechanistic Emphasis (Audit-Safe Summary)	Key Supporting Source(s)
Baseline clinical framing (as summarized in modern reviews)	Predominantly monocular functional deficit	BCVA	Modern reviews describe the historical clinical approach as largely centered on monocular acuity loss and penalization of the fellow eye, with BCVA commonly treated as the dominant endpoint in clinical reporting.	[1,2]
Plasticity framing (developmental)	Sensitive/critical-period plasticity disorder	BCVA + age/timing	Critical/sensitive-period concepts emphasize developmental constraints on recovery and motivate age/timing as clinically relevant modifiers of expected treatment response.	[4,34]
Binocular reframing (modern clinical + trials)	Binocular imbalance/suppression construct	Binocular measures (e.g., suppression) alongside BCVA	Abnormal binocular interactions (including interocular suppression) can be operationalized and quantified using dichoptic paradigms, supporting binocular endpoints alongside acuity in modern trials/syntheses.	[1,7,23,24,25,35]
Broader multi-domain framing (functional phenotype emphasis)	Multi-domain phenotype	Multi-metric outcomes (BCVA + binocular + functional impact)	Contemporary evidence emphasizes functional impact beyond acuity (e.g., reading-related limitation; broader daily-life functioning/HRQoL), and standardization efforts support adopting core outcome sets to improve comparability across studies.	[8,31,36,37]

**Table 3:** COSAMS -aligned core reporting matrix for amblyopia studies (children and adults), with additional mechanism-relevant variables recommended when feasible.

Domain	Variable	Recommended Metric	Core/Recommended	Rationale (Evidence-Anchored)
Demographics	Age	Years (continuous)	Core	Developmental/sensitive-period frameworks motivate age-stratified reporting and interpretation of response variability [4,34].
Etiology	Primary etiology (as defined by study) anisometropic/strabismic/combined; deprivation (if applicable)	Categorical	Core	Foundational phenotype descriptor for stratified reporting and interpretation across cohorts and treatment approaches [1,29].
Refraction	Spherical equivalent and cylinder (both eyes)	D (sphere/cyl; specify cycloplegia status and method)	Core	Minimum refractive reporting element aligned with COSAMS amblyopia core outcomes [31].
Acuity	BCVA amblyopic eye (distance and near)	logMAR (specify charts, distance, scoring)	Core	Core visual endpoint in COSAMS amblyopia reporting framework [31].
Acuity	BCVA fellow eye (distance and near)	logMAR	Core	Provides fellow-eye baseline context and enables derivation of interocular acuity difference (IOD) [31].
Binocular	Binocular function (e.g., stereoacuity)	Arcsec (report test/method; log-transform if analyzed)	Recommended (if measurable)	Binocular status is relevant for deep phenotyping and interpretation, particularly when interventions target binocular/dichoptic mechanisms [29,31].
Suppression	Quantified suppression index (method specified)	Continuous value (report scale/units); report “not measurable” explicitly	Recommended; particularly for binocular/dichoptic interventions	Pediatric methods enable quantified suppression indices; interpretation is method-dependent and should include transparent reporting of missingness/not measurable values [29,38].
Functional	Reading-related metric/sustained-task functional endpoint (when relevant)	Continuous (define endpoint and protocol)	Recommended	Functional limitation beyond BCVA is supported by reading-related and broader functional/QoL impact evidence [8,36,37].
Treatment descriptor	Optical phase (if used): duration and criteria	Weeks/months; specify stabilization/decision criteria	Core (if applicable)	Documents a baseline modifier when an optical treatment phase is included before additional therapy, supporting attribution of subsequent gains [39].
Treatment descriptor	Intervention class	Optical correction only/patching/atropine /graded optical penalization (e.g., Bangerter)/binocular –dichoptic–digital/VR/ perceptual learning/vision therapy–orthoptics/cone-biased spectral neuromodulation (research context only)	Core	Ensures interpretability across heterogeneous modalities in contemporary syntheses and supports mechanism-aligned interpretation across intervention classes [29,31,40,41,42,48,49,50,51,52].
Dose/adherence/ exposure	Prescribed dose and duration; adherence/compliance (and exposure parameters when relevant)	Hours/day + weeks/months; adherence metric specified; for spectral/light protocols report exposure duration/timing and verification parameters	Core	COSAMS includes adherence/compliance reporting as a core element; dose/adherence reporting supports comparability, and exposure specification is required for reproducible protocol reporting in light/filter paradigms [31].
Safety	Adverse events	Pre-specified AE list + counts	Core (when applicable)	COSAMS includes adverse events as a shared core outcome [31].
Patient-centred outcomes	Treatment burden/treatment impact (pediatric)	Validated PROM; instrument specified (name/version/ language/domains)	Recommended	PROM syntheses support instrument selection; psychological impact evidence supports measuring burden/impact [43,44].
Patient-centred outcomes	Long-term impact/QoL (adult/long-term)	AmbQoL total + domains (or validated alternative; instrument specified)	Recommended	Amblyopia-specific QoL instrument supports measurement of patient-perceived long-term impact [45].

**Note**: Core = recommended for all COSAMS-aligned studies to enable cross-study comparability. Recommended items should be included when feasible/measurable and are particularly relevant when interventions target binocular/dichoptic or training-based mechanisms.

## Data Availability

Data is contained within the article.

## Abbreviations

AAO	American Academy of Ophthalmology
BCVA	best-corrected visual acuity
COSAMS	Core Outcome Set for Amblyopia and Strabismus
DEM	Developmental Eye Movement (test)
EEG	electroencephalography
fMRI	functional magnetic resonance imaging
IOD	interocular acuity difference
logMAR	logarithm of the minimum angle of resolution
MEDLINE	Medical Literature Analysis and Retrieval System Online
MEG	magnetoencephalography
MRI	magnetic resonance imaging
PEDIG	Pediatric Eye Disease Investigator Group
PRISMA	Preferred Reporting Items for Systematic Reviews and Meta-Analyses
PROM	patient-reported outcome measure
QoL	quality of life
RCT	randomized controlled trial
rs-fMRI	resting-state functional magnetic resonance imaging
TVPS-3	Test of Visual-Perceptual Skills, Third Edition
V1	primary visual cortex
VA	visual acuity
VEP	visual evoked potential
VMI-6	Beery–Buktenica Developmental Test of Visual-Motor Integration, Sixth Edition

## References

[1] Levi D.M. (2020). Rethinking amblyopia. Vis. Res..

[2] Astle A.T., McGraw P.V. (2016). Amblyopia: Past, present and future. Ophthalmic Physiol. Opt..

[3] Çakır B., Özkan Aksoy N., Özmen S., Bursalı Ö. (2021). The effect of amblyopia on clinical outcomes of children with astigmatism. Ther. Adv. Ophthalmol..

[4] Holmes J.M., Levi D.M. (2018). Treatment of amblyopia as a function of age. Vis. Neurosci..

[5] Fu Z., Hong H., Su Z., Lou B., Pan C.W., Liu H. (2020). Global prevalence of amblyopia and disease burden projections through 2040: A systematic review and meta-analysis. Br. J. Ophthalmol..

[6] Thompson B., Morrone M.C., Bex P., Lozama A., Sabel B.A. (2024). Harnessing brain plasticity to improve binocular vision in amblyopia: An evidence-based update. Eur. J. Ophthalmol..

[7] Birch E.E., Morale S.E., Jost R.M., De La Cruz A., Kelly K.R., Wang Y.Z., Bex P.J. (2016). Assessing suppression in amblyopic children with a dichoptic eye chart. Investig. Ophthalmol. Vis. Sci..

[8] Birch E.E., Kelly K.R. (2017). Pediatric ophthalmology and childhood reading difficulties: Amblyopia and slow reading. J. AAPOS.

[9] Hernández-Andrés R., Serrano M.Á., Alacreu-Crespo A., Luque M.J. (2025). Randomised trial of three treatments for amblyopia: Vision therapy and patching, perceptual learning and patching alone. Ophthalmic Physiol. Opt..

[10] Black A.A., Wood J.M., Hoang S., Thomas E., Webber A.L. (2021). Impact of Amblyopia on Visual Attention and Visual Search in Children. Investig. Ophthalmol. Vis. Sci..

[11] Rakshit A., Majhi D., Schmid K.L., Warkad V., Atchison D.A., Webber A.L. (2024). Fine Motor Skills, Reading Speed, and Self-Reported Quality of Life in Adults With Amblyopia and/or Strabismus. Investig. Ophthalmol. Vis. Sci..

[12] Li T., Qureshi R., Taylor K. (2019). Conventional occlusion versus pharmacologic penalization for amblyopia. Cochrane Database Syst. Rev..

[13] Yazdani N., Sadeghi R., Momeni-Moghaddam H., Zarifmahmoudi L., Ehsaei A., Barrett B.T. (2017). Part-time versus full-time occlusion therapy for treatment of amblyopia: A meta-analysis. J. Curr. Ophthalmol..

[14] Kelly K.R., Jost R.M., Dao L., Beauchamp C.L., Leffler J.N., Birch E.E. (2016). Binocular iPad game vs. patching for treatment of amblyopia in children: A randomized clinical trial. JAMA Ophthalmol..

[15] Holmes J.M., Manh V.M., Lazar E.L., Beck R.W., Birch E.E., Kraker R.T., Crouch E.R., Erzurum S.A., Khuddus N., Summers A.I. (2016). Effect of a binocular iPad game vs. part-time patching in children aged 5 to 12 years with amblyopia: A randomized clinical trial. JAMA Ophthalmol..

[16] Wygnanski-Jaffe T., Kushner B.J., Moshkovitz A., Belkin M., Yehezkel O. (2023). An eye-tracking-based dichoptic home treatment for amblyopia: A multicenter randomized clinical trial. Ophthalmology.

[17] Wygnanski-Jaffe T., Kushner B.J., Moshkovitz A., Belkin M., Yehezkel O. (2025). High-adherence dichoptic treatment versus patching in anisometropic and small angle strabismus amblyopia: A randomized controlled trial. Am. J. Ophthalmol..

[18] Jost R.M., Birch E.E., Wang Y.Z., Dao L.M., Stager D., Luu B., Beauchamp C.L., Giridhar P., Brin T.A., Baldwin A.S. (2024). Patch-free streaming contrast-rebalanced dichoptic cartoons versus patching for treatment of amblyopia in children aged 3 to 5 years: A pilot randomized clinical trial. J. AAPOS.

[19] Jost R.M., Hudgins L.A., Dao L.M., Stager D.R., Luu B., Beauchamp C.L., Hunter J.S., Giridhar P., Wang Y.Z., Birch E.E. (2022). Randomized clinical trial of streaming dichoptic movies versus patching for treatment of amblyopia in children aged 3 to 7 years. Sci. Rep..

[20] Xiao S., Angjeli E., Wu H.C., Gaier E.D., Gomez S., Travers D.A., Binenbaum G., Langer R., Hunter D.G., Repka M.X. (2022). Randomized controlled trial of a dichoptic digital therapeutic for amblyopia. Ophthalmology.

[21] Manny R.E., Holmes J.M., Kraker R.T., Li Z., Waters A.L., Kelly K.R., Kong L., Crouch E.R., Lorenzana I.J., Alkharashi M.S. (2022). A randomized trial of binocular Dig Rush game treatment for amblyopia in children aged 4 to 6 years. Optom. Vis. Sci..

[22] Meqdad Y., El-Basty M., Awadein A., Gouda J., Hassanein D. (2024). Randomized controlled trial of patching versus dichoptic stimulation using virtual reality for amblyopia therapy. Curr. Eye Res..

[23] Pineles S.L., Aakalu V.K., Hutchinson A.K., Galvin J.A., Heidary G., Binenbaum G., VanderVeen D.K., Lambert S.R. (2020). Binocular treatment of amblyopia: A report by the American Academy of Ophthalmology. Ophthalmology.

[24] Roda M., Pellegrini M., Di Geronimo N., Vagge A., Fresina M., Schiavi C. (2021). Binocular treatment for amblyopia: A meta-analysis of randomized clinical trials. PLoS ONE.

[25] Chen C.W., Zhu Q., Duan Y.B., Yao J.Y. (2021). Comparison between binocular therapy and patching for treatment of amblyopia: A meta-analysis of randomised controlled trials. BMJ Open Ophthalmol..

[26] Brin T.A., Chow A., Carter C., Oremus M., Bobier W., Thompson B. (2021). Efficacy of vision-based treatments for children and teens with amblyopia: A systematic review and meta-analysis of randomised controlled trials. BMJ Open Ophthalmol..

[27] Tang A.C., Wang X., Yang W.J., Guo J.L., Li Y.L., Yang T.Y., An Z., Reynaud A., Liu L.Q. (2025). Comparison between dichoptic and monocular training protocols in the treatment of unilateral amblyopia: An individual participant data meta-analysis. Ophthalmic Epidemiol..

[28] Ming X., Huang G., Chen X., Liao M., Liu L. (2025). A systematic review and meta-analysis of perceptual learning and video game training for adults with monocular amblyopia. Ophthalmol. Ther..

[29] Meier K., Tarczy-Hornoch K. (2022). Recent treatment advances in amblyopia. Annu. Rev. Vis. Sci..

[30] Martínez-Pérez C., Oliveira A.P. (2026). Systematic review and meta-analysis of RCTs on efficacy of conventional vs. emerging treatments for amblyopia. Life.

[31] Al-Jabri S., Rowe F.J., Kirkham J.J. (2021). Core outcome set for three ophthalmic conditions: A healthcare professional and patient consensus on core outcome sets for amblyopia, ocular motility and strabismus (COSAMS Study). BMJ Open.

[32] Kiorpes L. (2016). The puzzle of visual development: Behavior and neural limits. J. Neurosci..

[33] Atkinson J. (2017). The Davida Teller Award Lecture, 2016: Visual brain development: A review of dorsal stream vulnerability and its clinical implications. J. Vis..

[34] Hensch T.K., Quinlan E.M. (2018). Critical periods in amblyopia. Vis. Neurosci..

[35] Hamm L.M., Chen Z., Li J., Black J., Dai S., Yuan J., Yu M., Thompson B. (2017). Interocular suppression in children with deprivation amblyopia. Vis. Res..

[36] Kelly K.R., Pang Y., Thompson B., Niechwiej-Szwedo E., Drews-Botsch C.D., Webber A.L. (2025). Functional consequences of amblyopia and its impact on health-related quality of life. Vis. Res..

[37] Birch E.E., Kelly K.R. (2023). Amblyopia and the whole child. Prog. Retin. Eye Res..

[38] Chen H., He Z., Xu J., Mao Y., Liang Y., Lin D., Xu M., Dai Z., Chen X., Zhou J. (2019). A convenient and robust test to quantify interocular suppression for children with amblyopia. i-Perception.

[39] Proudlock F.A., Hisaund M., Maconachie G., Papageorgiou E., Manouchehrinia A., Dahlmann-Noor A., Khandelwal P., Self J., Beisse C., Gottlob I. (2024). Extended optical treatment versus early patching with an intensive patching regimen in children with amblyopia in Europe (EuPatch): A multicentre, randomised controlled trial. Lancet.

[40] Castro-Torres J.J., Martino F., Casares-López M., Ortiz-Peregrina S., Ortiz C. (2021). Visual performance after the deterioration of retinal image quality: Induced forward scattering using Bangerter foils and fog filters. Biomed. Opt. Express.

[41] Zhang P., Wang H.L., Ren W.C., Guo H., Yang J., Tao J., Yang Z., Li Y., Chen L., Zhang Y. (2022). The effect of Bangerter filters on visual acuity and contrast sensitivity with external noise. Front. Neurosci..

[42] Jiang J., Zhao T., Yin Y., Han M., Wu X., Chen Y. (2025). Bangerter filter’s role in regulating ocular axial length (anisometropic amblyopia treatment). BMC Ophthalmol..

[43] Kumaran S.E., Khadka J., Baker R., Pesudovs K. (2018). Patient-reported outcome measures in amblyopia and strabismus: A systematic review. Clin. Exp. Optom..

[44] Haine L., Taylor I., Vaughan M. (2025). The psychological impact of amblyopia treatment: A systematic literature review. Br. Ir. Orthopt. J..

[45] Webber A.L., Wood J. (2005). Amblyopia: Prevalence, natural history, functional effects and treatment. Clin. Exp. Optom..

[46] Gu L., Wang Y., Feng L., Li S., Zhang M., Ye Q., Zhuang Y., Lu Z.L., Li J., Yuan J. (2021). Meridian-Specific and Post-Optical Deficits of Spatial Vision in Human Astigmatism: Evidences From Psycho-Physical and EEG Scalings. Front. Psychol..

[47] Yap T.P., Luu C.D., Suttle C.M., Chia A., Boon M.Y. (2024). The development of meridional anisotropies in neurotypical children with and without astigmatism: Electrophysiological and psychophysical findings. Vis. Res..

[48] Ma M.M.L., Kang Y., Scheiman M., Chen Q., Ye X., Pan L., Deng J., Su G., Zhang G., Chen X. (2024). Office-based vergence and anti-suppression therapy for the treatment of small-to-moderate angle intermittent exotropia: A randomised clinical trial. Ophthalmic Physiol. Opt..

[49] Ma M.M.L., Kang Y., Scheiman M., Chen Q., Ye X., Pan L., Deng J., Su G., Zhang G., Chen X. (2025). Effect of office-based vergence and anti-suppression therapy on binocular vision and accommodation in small-to-moderate angle intermittent exotropia: A randomised clinical trial. Ophthalmic Physiol. Opt..

[50] Ibrahimi D., García-Martínez J.R. (2025). Cone-Specific Filter-Based Neuromodulation: A Proposed Clinical Framework for Amblyopia, Strabismus, and ADHD. Clin. Pract..

[51] Ibrahimi D., Mendiola-Santibañez J.D., Cruz-Martínez E., Rodríguez-Reséndiz J., Pacheco I.T. (2021). Cortical activity at baseline and during light stimulation in patients with strabismus and amblyopia. IEEE Access.

[52] Ibrahimi D., Mendiola-Santibañez J.D., Cruz-Martínez E., Gómez-Espinosa A., Torres-Pacheco I. (2021). Changes in brain activity and visual performance after a complete cycle of light therapy. Brain Sci..

[53] Mitchell D.E., Maurer D. (2022). Critical periods in vision revisited. Annu. Rev. Vis. Sci..

[54] Rakshit A., Schmid K.L., Webber A.L. (2024). Fine visuomotor skills in amblyopia: A systematic review and meta-analysis. Br. J. Ophthalmol..

[55] Sá C.D.S.C., Luz C., Pombo A., Rodrigues L.P., Cordovil R. (2021). Motor Competence in Children With and Without Amblyopia. Percept. Mot. Skills.

[56] Wei C., Yang D.P., Yang Y., Yang W.H., Lu Y.M., Yu X.P., Chang S. (2024). Visual and auditory attention defects in children with intermittent exotropia. Ital. J. Pediatr..

[57] Kelly K.R., Nouradanesh M., Jost R.M., Cheng-Patel C.S., Birch E.E., Wang S.X., Tung J.Y., Niechwiej-Szwedo E. (2025). Eye-hand coordination during visually-guided reaching in children with monocular deprivation amblyopia. Vis. Res..

[58] Argilés M., Gispets J., Lupón N., Sunyer-Grau B., Rovira-Gay C., Pérez-Ternero M., Berta-Cabañas M. (2023). Impact of strabismus and binocular dysfunctions in the developmental eye movement test and test of visual perception skills: A multicentric and retrospective study. J. Optom..

[59] Ibrahimi D., Mendiola-Santibañez J.D., Gkaros A.P. (2021). Analysis of the potential impact of strabismus with and without amblyopia on visual-perceptual and visual-motor skills evaluated using TVPS-3 and VMI-6 tests. J. Optom..

[60] Mao D., Liu C., Yin Z., Cui Z., Zhang J., Li X., Huang Y., Chen H., Bao J. (2025). Effects of anisometropic amblyopia on visual cognitive functions in children. Clin. Exp. Ophthalmol..

[61] Nagarajan K., Luo G., Narasimhan M., Satgunam P. (2022). Children With Amblyopia Make More Saccadic Fixations When Doing the Visual Search Task. Investig. Ophthalmol. Vis. Sci..

[62] Atiya A., Mani R., Webber A., Narayanan A., Khuu S.K. (2026). Saccadic eye movements in amblyopia: A systematic review and meta-analysis. Clin. Exp. Optom..

[63] Ming X., Huang G., Liao M., Jiang P., Liu L. (2025). Psychophysical assessment of face perception deficits in adults with amblyopia through top-down and bottom-up visual processing pathways. Front. Neurosci..

[64] Jayakaran P., Aman W., Fernando U., Hackfath K., McPherson A., Williams M., Mitchell L. (2021). Sensory organization for postural control in children with strabismus—A systematic review and meta-analysis. Gait Posture.

[65] Peng J., Yao F., Li Q., Ge Q., Shi W., Su T., Tang L., Pan Y., Liang R., Zhang L. (2021). Alternations of interhemispheric functional connectivity in children with strabismus and amblyopia: A resting-state fMRI study. Sci. Rep..

[66] Shi Y.D., Ge Q.M., Lin Q., Liang R.B., Li Q.Y., Shi W.Q., Li B., Shao Y. (2022). Functional connectivity density alterations in children with strabismus and amblyopia based on resting-state functional magnetic resonance imaging (fMRI). BMC Ophthalmol..

[67] Wang H., Crewther S., Liang M., Laycock R., Yu T., Alexander B., Crewther D., Wang J., Yin Z. (2017). Impaired activation of visual attention network for motion salience is accompanied by reduced functional connectivity between frontal eye fields and visual cortex in strabismic amblyopia. Front. Hum. Neurosci..

[68] Li H., Li W., Hong J., Liu J., Hao J., Dai W., Liu Z., Fu J. (2024). Altered functional connectivity of resting-state networks and the correlation with clinical characteristics in intermittent exotropia adult patients: A resting-state magnetic resonance imaging study. BMC Ophthalmol..

[69] Wang Y., Wu Y., Luo L., Li F. (2023). Structural and functional alterations in the brains of patients with anisometropic and strabismic amblyopia: A systematic review of magnetic resonance imaging studies. Neural Regen. Res..

[70] Mu Y., Qin X., Yao N., Zhang D., Yang Y., Huang Z., Chen F., Liu C., Dong Y., Zhang R. (2022). Single center to evaluate and compare anisometropic amblyopia in adults using blood oxygenation level-dependent functional magnetic resonance imaging and diffusion kurtosis imaging. Med Sci. Monit..

[71] Hou C., Tyson T.L., Uner I.J., Nicholas S.C., Verghese P. (2021). Excitatory contribution to binocular interactions in human visual cortex is reduced in strabismic amblyopia. J. Neurosci..

[72] Julku H., Rouhinen S., Huttunen H.J., Lindberg L., Liinamaa J., Saarela V., Karvonen E., Booms S., Mäkelä J.P., Uusitalo H. (2021). Reduced evoked activity and cortical oscillations are correlated with anisometropic amblyopia and impairment of visual acuity. Sci. Rep..

[73] Dahal M., Dahal H.N., Gautam P., Shrestha J.B., Khanal S. (2023). Pattern visual evoked potential and foveal sensitivity in amblyopia. Doc. Ophthalmol..

[74] Yap T.P., Luu C.D., Suttle C.M., Chia A., Boon M.Y. (2021). Characterising the orientation-specific pattern-onset visual evoked potentials in children with bilateral refractive amblyopia and non-amblyopic controls. Doc. Ophthalmol..

